# Restoration of Anterior Teeth Defect With Porcelain Laminate Veneer Using the Refractory Technique: A Case Report

**DOI:** 10.7759/cureus.67501

**Published:** 2024-08-22

**Authors:** Trang T Nguyen, Anh Nguyen Viet, Loan T Nguyen

**Affiliations:** 1 Dentistry, Phenikaa University, Hanoi, VNM; 2 Dentistry, Viet Anh Orthodontic Clinic, Hanoi, VNM

**Keywords:** cosmetic dentistry, preservative approach, ultrathin veneer, refractory die, ceramic veneer

## Abstract

Anterior teeth play an important role in the aesthetic appearance of an individual. Hence, restoration of the anterior teeth has always been a significant concern for both dentists and patients worldwide. Indirect restoration using laminate veneers has revolutionized cosmetic dentistry by minimizing tooth preparation compared to full dental crowns. It also enhances vitality, color matching, and superior mechanical properties compared to direct composite restoration. The success of veneer restoration depends on case selection, material choice, and fabrication technique, tailored for every patient based on a thorough consideration of the existing lesion and the needs of the patient. This clinical report exemplifies the conservative treatment of a refractory defect on the labial surface of the maxillary central incisor using a minimal-thickness veneer fabricated from a refractory porcelain system.

## Introduction

Anterior teeth play an important role in the aesthetic appearance of an individual. Therefore, restoring the anterior teeth has always been a major concern for both dentists and patients worldwide. Among the various approaches available to restore tooth defects, laminate veneers have drawn significant attention due to their minimally invasive nature. Preparation of ceramic veneers resulted in 3% to 30% of the coronal tooth structure reduction, while about 63% to 72% of the coronal tooth structure reduction was expected for full-coverage ceramic crowns [1]. Compared to direct composite restorations, ceramic veneers generally have longer longevity, superior mechanical strength, and better control over optical properties [2].

There are numerous ceramic materials available for dental restorations, including traditional glass-based feldspathic porcelain, crystallized lithium disilicate, leucite-reinforced feldspathic porcelain, lithium disilicate reinforced with zirconia, and polycrystalline zirconia. Among these, feldspathic ceramic has been the material of choice for aesthetic restorations due to its excellent optical properties and its ability to replicate the natural color of teeth, thanks to its glassy matrix. The long-term survival rate, acceptable mechanical strength, and superior aesthetic results of feldspathic ceramics have been well documented in the literature [2,3]. While lithium ceramics also offer favorable aesthetic outcomes, they are less translucent due to their crystalline component. In terms of strength, lithium ceramics are superior because of this added crystalline content, whereas feldspathic ceramics are relatively weaker. However, for restorations of anterior teeth, which are less subject to masticatory forces, particularly on the outer surfaces of incisors, feldspathic ceramics still provide sufficient hardness and long-term survival [3].

Continuous advancements in the fracture strength of glass ceramics and adhesive systems, combined with the growing demand for more aesthetic solutions, have led to the increasing popularity of minimally invasive ceramic veneers. Ultrathin veneers can be fabricated using various methods, such as die-casting, ceramic pressing, or computer-aided design and computer-aided manufacturing (CAD/CAM) technology. The direct ceramic layering method on a refractory die is the traditional technique for fabricating porcelain veneers. Although it requires high technical skill, this method allows for the creation of restorations with very thin margins, making it especially suitable for unprepared or minimally prepared veneers. Various clinical case reports and long-term studies have demonstrated the success of minimal or no-preparation feldspathic veneers produced using the refractory die technique. These studies have shown that veneers can be as thin as 0.5 mm or less while maintaining sufficient physical strength when supported by a resin-based luting agent, thereby enhancing the overall performance and longevity of the restoration [4,5].

## Case presentation

A 32-year-old female patient presented to our clinic with the chief complaint of an unaesthetic left central incisor. The patient had previously had the facial surface defect of this tooth corrected several times using direct composite resin restorations, but they fell out.

Treatment planning

**Figure 1:**
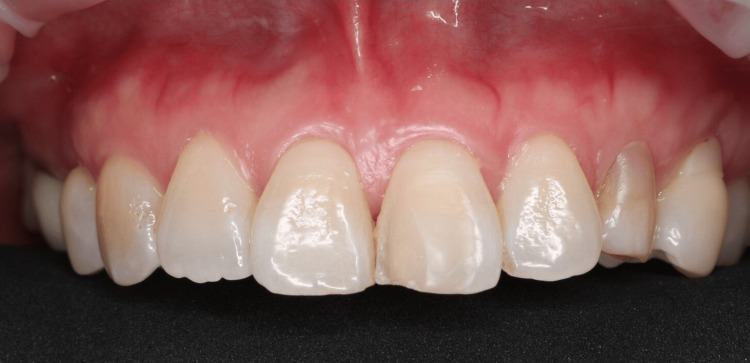
Pre-operative intraoral photograph showing a defect in the labial surface of the left central incisor, confined to the enamel layer

The patient's medical history was unremarkable, with no history of bruxism or tooth clenching. An intraoral examination revealed a defect on the buccal surface of the left central incisor, confined to the enamel layer (Figure 1).

Various treatment options, along with their advantages and disadvantages, were discussed, including full ceramic crowns and indirect laminate veneers. The patient opted for the less invasive ceramic veneer option. Proper consent was obtained before proceeding with treatment. The patient received dental prophylaxis and oral hygiene instructions. A study model and diagnostic mock-up were created using light-cure composite resin to evaluate the configuration and thickness of the future prosthesis. Upon investigation, it was determined that a ceramic veneer fabricated using the refractory die technique, with no tooth preparation, was indicated.

Tooth preparation

**Figure 2:**
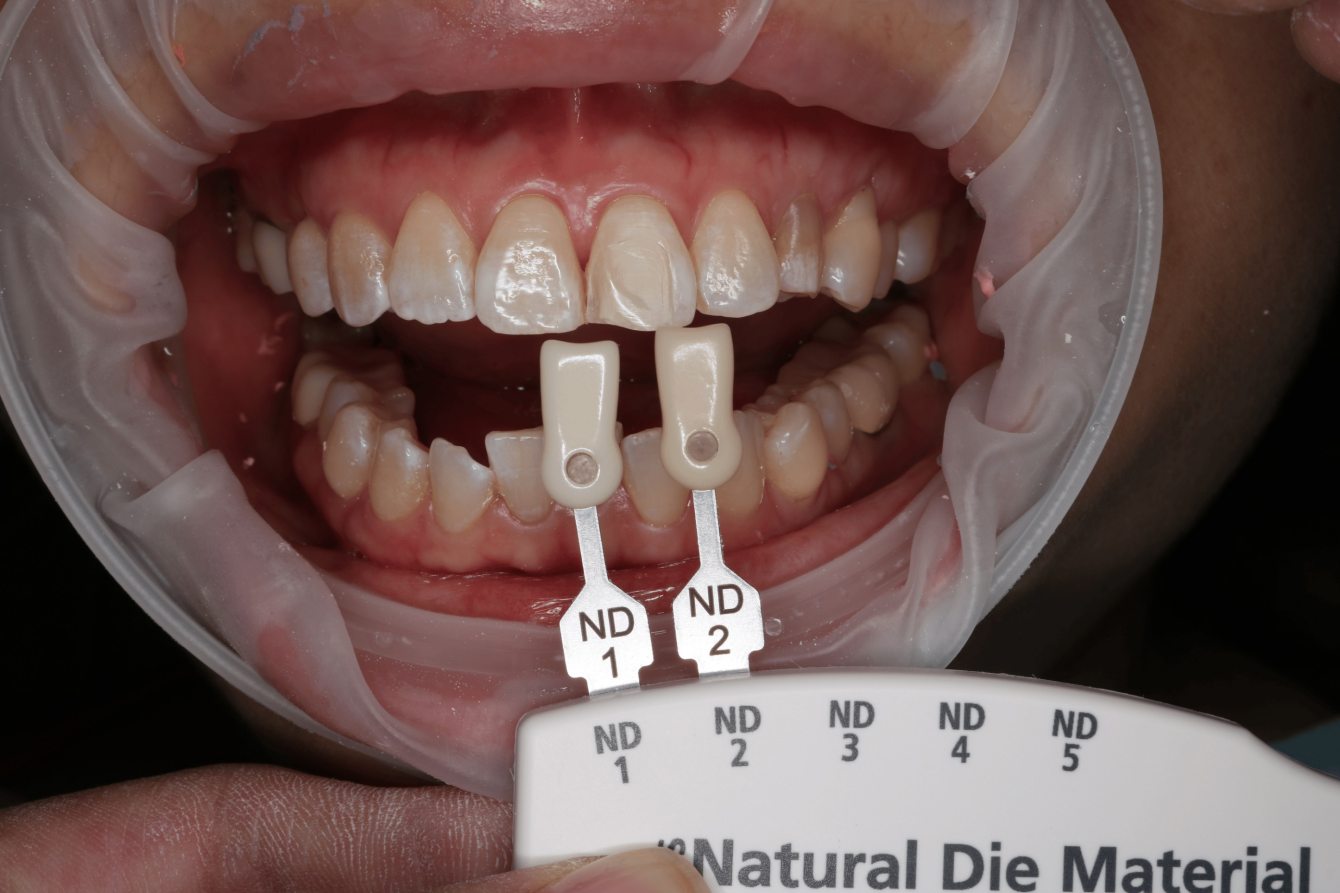
The shade of the minimally prepared tooth was selected using a visual stump shade guide to ensure an accurate color match for the restoration

As the defect was confined to the enamel layer, no further tooth preparation was performed except for rounding off sharp margins and removing any undercuts. The defect was smoothed and polished to improve the adaptation of the restoration. The shade of prepared and adjacent teeth was then selected (IPS natural die material shade guide, Ivoclar, Germany) (Figure 2).

Impression

An impression of the defective tooth was taken using silicone putty (Elite P&P, Zhermack, Italy) on a stock tray.

Provisional restoration

After taking the impression, the defective surface was temporarily restored with a light-curing composite (3M™ Filtek™ Z350XT Flowable, 3M, USA) to protect the tooth from saliva, prevent hypersensitivity, and maintain aesthetics.

Fabrication of the veneer

**Figure 3:**
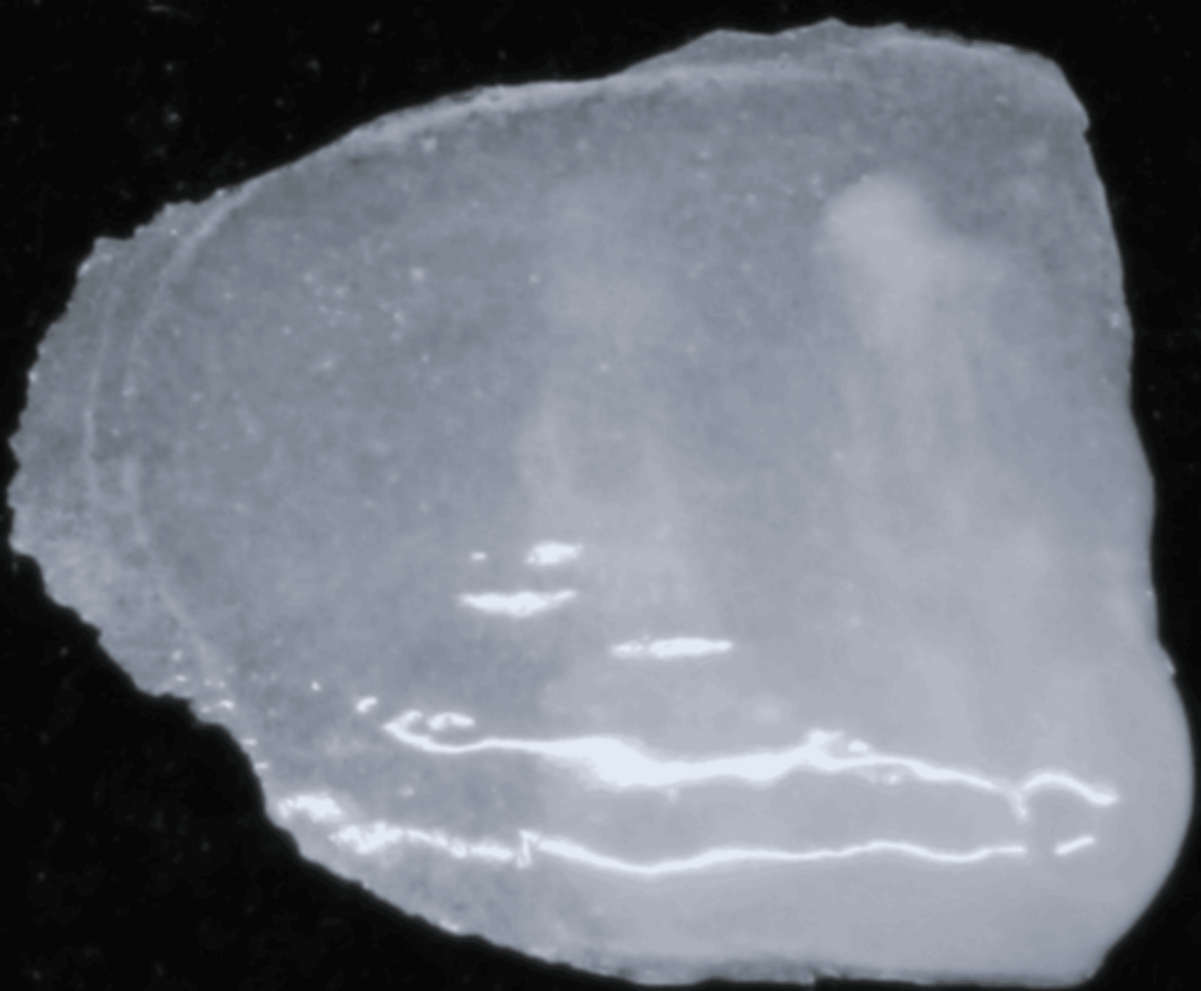
Ultra-thin veneer fabricated from feldspathic porcelain was refined and adjusted before being delivered to the dental office

The master model was poured using type IV gypsum (Elite Rock, Zhermack, Italy), and a diagnostic mock-up was made with a light-curing composite. A refractory die was duplicated from the master model. Low-fusing feldspathic ceramic (IPS e.max Ceram, Ivoclar, Germany) was applied to the die using the layering technique. The veneer was then divested and adjusted on the die to refine the shape, confirm marginal fidelity, and ensure surface smoothness (Figure 3).

Cementation

**Figure 4:**
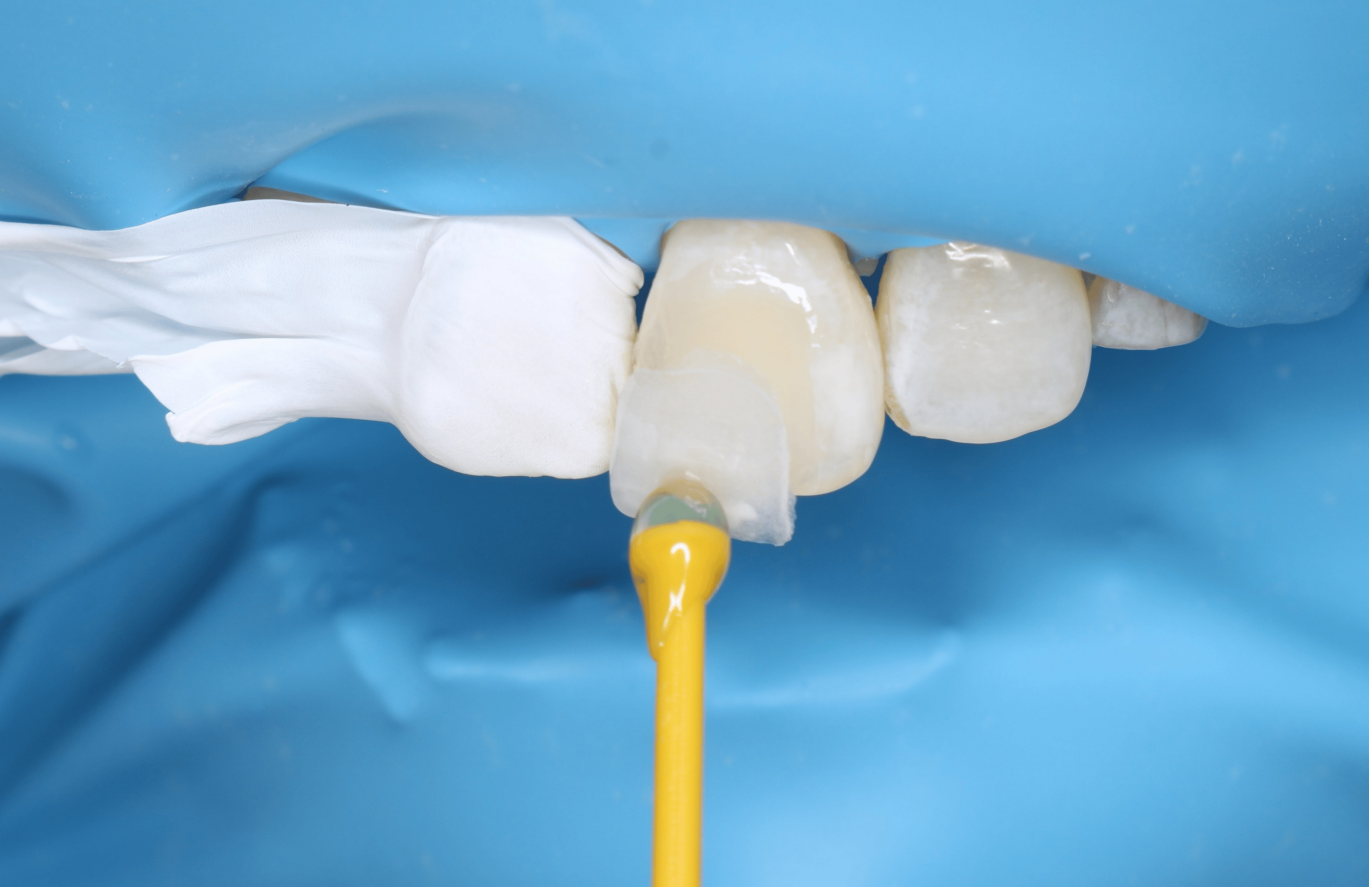
Intraoral view during the try-in and cementation process of the veneer, ensuring proper fit and alignment The cementation was performed under moisture-free condition with rubber dam isolation.

The shade match, configuration, and marginal fit of the veneer were checked intraorally. Once everything was satisfactory, the cementation was carried out. The final restoration was cemented with a clear resin light-cured luting agent (Variolink, Ivoclar-Vivadent, Amherst, NY, USA). First, the intaglio surface of the veneer was etched with 5% hydrofluoric acid for 60 seconds (IPS® Ceramic etching gel, Ivoclar, USA). After post-etching cleaning, the veneer was dried and primed with a silane coupling agent for 20 seconds before dispersing with oil-free air (Monobond Plus, Ivoclar, USA). The tooth surface was cleaned with fluoride-free pumice powder and then etched with 37% phosphoric acid (Total Etch, Ivoclar, USA) for 15 seconds. A bonding agent (Adhese Universal, Ivoclar, USA) was applied for 20 seconds after washing and air drying to evaporate the solvent, followed by light-curing for 10 seconds (Woodpecker LED.F, China). The veneer was bonded with light-polymerizing resin cement (Variolink Esthetic LC, Ivoclar, USA). Excess cement was removed from the margins with an explorer, and excess interproximal cement was removed with dental floss. Light polymerization was performed for 20 seconds. The veneer was then covered with glycerin gel as an oxygen barrier to ensure better polymerization of the resin cement. Then light polymerization was carried out for 20 seconds on each margin (gingival, incisal edge, proximal). After final marginal polishing, occlusion was checked for any disturbances. The procedure was performed under moisture-free conditions using rubber dam isolation (Figure 4).

**Figure 5:**
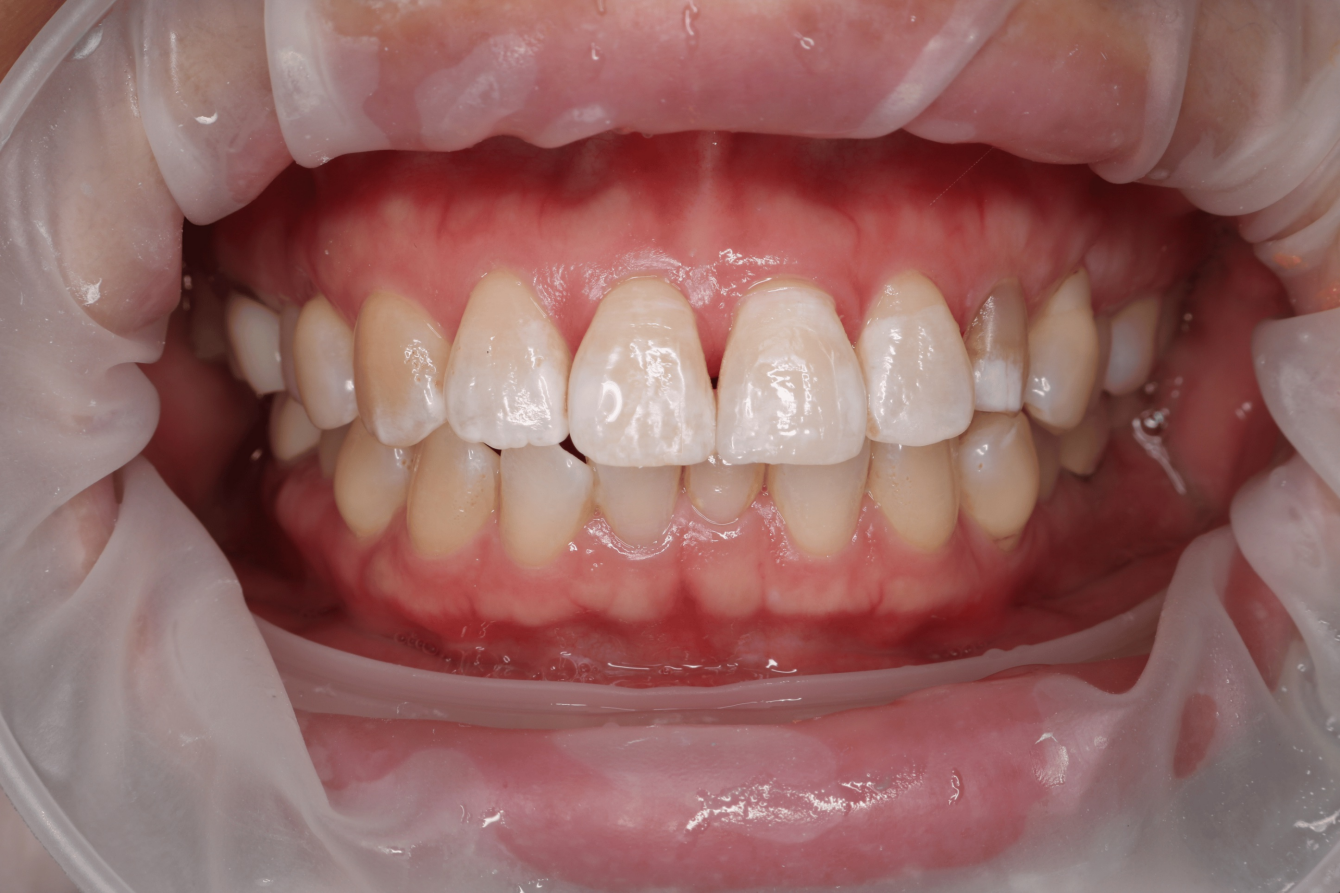
Post-operative intraoral photograph of the restored left central incisor taken immediately after veneer placement, illustrating the aesthetic integration with indiscernible tooth-restoration margin

**Figure 6:**
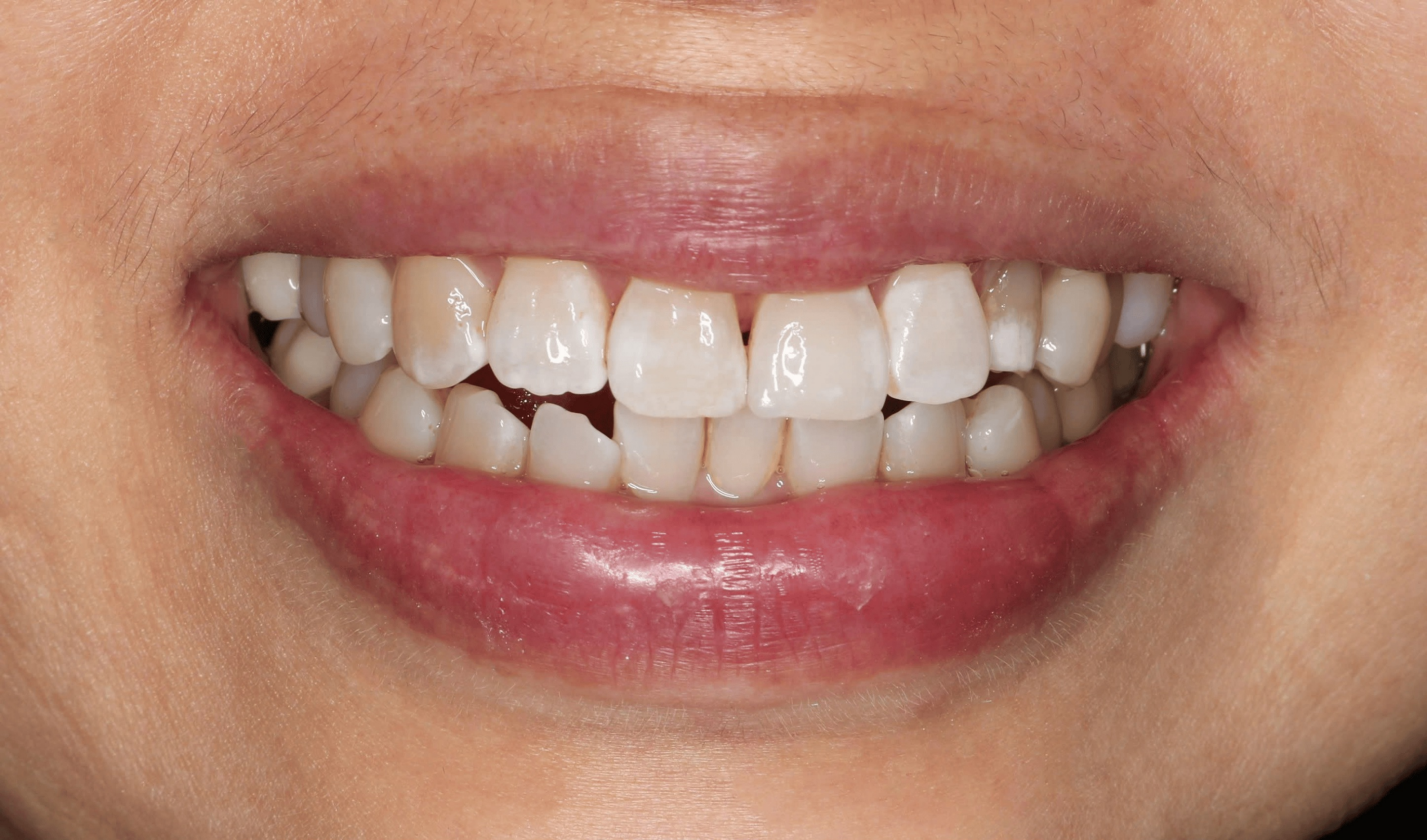
Post-operative extraoral photograph showing the final appearance of the left central incisor after veneer restoration, highlighting the natural look and harmony with surrounding teeth

Postoperative intraoral extraoral photographs of the patient were shown in Figures 5, 6, respectively.

**Figure 7:**
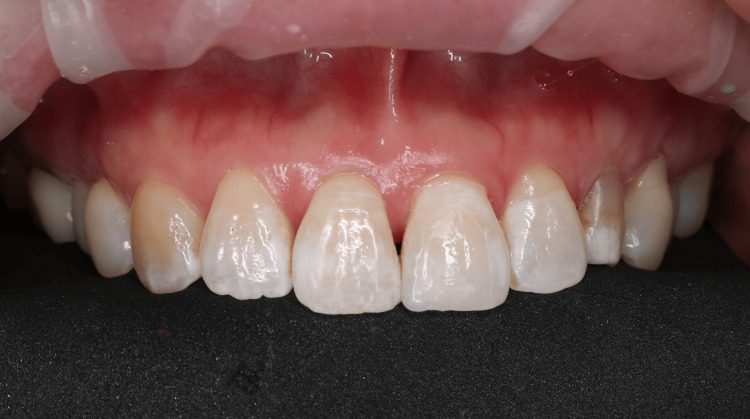
One-week after cementation The follow-up examination showed no signs of veneer dislodgement or marginal gaps.

The patient’s satisfaction was evaluated using a questionnaire developed by Sulaya and Guttal [6]. The patient provided an overall score of 5 out of 5 for all questions, indicating that she was very satisfied with the treatment. The patient returned for a follow-up examination one week later. The veneer remained securely in place, with no signs of marginal gaps or occlusal disturbances (Figure 7). The patient reported being satisfied with the results.

## Discussion

This case report illustrates the restoration of a central incisor defect using an ultra-thin laminate veneer fabricated via the refractory die technique, with no tooth preparation. The overall outcome was satisfactory in terms of color matching, marginal fit, and harmonious emergence profile.

While direct restorations remain a low-cost, viable option, they failed in this patient's case. As supported by scientific evidence, advances in dental ceramics and adhesive systems have enabled restorations with superior longevity and aesthetic outcomes [2]. The choice of a no-preparation approach was based on evaluating the existing defect, which was confined to the enamel layer. Further preparation could increase the restoration's thickness but would require more tooth structure removal. Additionally, such preparation might expose dentin, compromising the veneer’s long-term survival rate since current adhesive systems bond more effectively to enamel [7].

The diagnostic mock-up provided valuable information regarding the thickness and configuration of the future restoration. Given the minimal loss of dental structure and the thin margin of the restoration, an ultra-thin veneer made using the refractory die technique was chosen. This technique allows for the divesting of the veneer with minimal risk of marginal breakage and offers better control over the ceramic's color, resulting in a more natural appearance. Low-fusing feldspathic porcelain is not only suitable for the refractory technique but also yields excellent aesthetic results, recreating natural tooth color with seamless margins. Although the veneer finishing line was placed above the gingival margin, it was almost indiscernible, demonstrating the superior translucency of this ceramic. The 10-year survival rate of feldspathic porcelain bonded to enamel is also favorable, reaching as high as 95% [2].

However, this technique is time-consuming and requires a highly skilled technician. An alternative could be using castable ceramic with the heat-pressed technique. While feldspathic porcelain offers the best aesthetic control and optical properties, the heat-pressed technique is less technically demanding. The high translucency and low refractive index of lithium disilicate crystals allow for natural tooth color restoration [8,9]. In recent years, the introduction and rapid development of 3D printing technology have shown promising economic and mechanical advantages over traditional methods for fabricating dental laminate veneers. 3D printing does not require extensive manual work and is less dependent on the technician's skill level. The process is largely automated, relying on digital design and manufacturing techniques, which reduce the variability and human error associated with traditional methods. Although some studies support the feasibility of using 3D printing for thin to ultra-thin veneers, there is still a lack of comprehensive studies on the marginal fitness and mechanical performance of prostheses produced using this method. Furthermore, improvements in the digital workflow, optimal material ratios, and the propensity to develop defects during manufacturing still require further investigation [10,11].

## Conclusions

This case report demonstrates the successful restoration of a central incisor defect using an ultra-thin laminate veneer without tooth preparation. The minimally invasive approach, utilizing the refractory die technique with feldspathic porcelain, provided a highly aesthetic and functional outcome, effectively addressing the patient's esthetic concerns with excellent color matching and marginal fit. The success of this treatment emphasizes the importance of a customized treatment plan tailored to the unique needs and conditions of each patient. Additionally, the collaboration between the clinician and the dental laboratory on material choice and fabrication technique was crucial for the precise fabrication and application of the veneer. These factors collectively highlight the significance of personalized care and teamwork in achieving optimal results in cosmetic dentistry.
